# Drugs targeting the mitochondrial pore act as citotoxic and cytostatic agents in temozolomide-resistant glioma cells

**DOI:** 10.1186/1479-5876-7-13

**Published:** 2009-02-05

**Authors:** Annalisa Lena, Mariarosa Rechichi, Alessandra Salvetti, Barbara Bartoli, Donatella Vecchio, Vittoria Scarcelli, Rosina Amoroso, Lucia Benvenuti, Rolando Gagliardi, Vittorio Gremigni, Leonardo Rossi

**Affiliations:** 1 Dipartimento di Morfologia Umana e Biologia Applicata, University of Pisa, Via Volta 4, 56126 Pisa, Italy; 2 U.O. Neurochirurgia, ASL6, Livorno Hospital, Livorno, 57100, Italy; 3 Istituto Toscano Tumori, Florence, Italy

## Abstract

**Background:**

High grade gliomas are one of the most difficult cancers to treat and despite surgery, radiotherapy and temozolomide-based chemotherapy, the prognosis of glioma patients is poor. Resistance to temozolomide is the major barrier to effective therapy. Alternative therapeutic approaches have been shown to be ineffective for the treatment of genetically unselected glioma patients. Thus, novel therapies are needed. Mitochondria-directed chemotherapy is an emerging tool to combat cancer, and inner mitochondrial permeability transition (MPT) represents a target for the development of cytotoxic drugs. A number of agents are able to induce MPT and some of them target MPT-pore (MPTP) components that are selectively up-regulated in cancer, making these agents putative cancer cell-specific drugs.

**Objective:**

The aim of this paper is to report a comprehensive analysis of the effects produced by selected MPT-inducing drugs (Betulinic Acid, Lonidamine, CD437) in a temozolomide-resistant glioblastoma cell line (ADF cells).

**Methods:**

EGFRvIII expression has been assayed by RT-PCR. EGFR amplification and PTEN deletion have been assayed by differential-PCR. Drugs effect on cell viability has been tested by crystal violet assay. MPT has been tested by JC1 staining. Drug cytostatic effect has been tested by mitotic index analysis. Drug cytotoxic effect has been tested by calcein AM staining. Apoptosis has been assayed by Hoechst incorporation and Annexine V binding assay. Authophagy has been tested by acridine orange staining.

**Results:**

We performed a molecular and genetic characterization of ADF cells and demonstrated that this line does not express the EGFRvIII and does not show EGFR amplification. ADF cells do not show PTEN mutation but differential PCR data indicate a hemizygous deletion of PTEN gene. We analyzed the response of ADF cells to Betulinic Acid, Lonidamine, and CD437. Our data demonstrate that MPT-inducing agents produce concentration-dependent cytostatic and cytotoxic effects in parallel with MPT induction triggered through MPTP. CD437, Lonidamine and Betulinic acid trigger apoptosis as principal death modality.

**Conclusion:**

The obtained data suggest that these pharmacological agents could be selected as adjuvant drugs for the treatment of high grade astrocytomas that resist conventional therapies or that do not show any peculiar genetic alteration that can be targeted by specific drugs.

## Background

High grade gliomas, which include anaplastic gliomas (WHO grade III) and glioblastomas (GBM, WHO grade IV) are the most common types of primary brain tumor in adults. The prognosis for patients with this tumor is very poor, with most of them dying within 1 year after diagnosis [1]. With the current standard care – which consists of maximal surgical resection, concurrent radiation therapy and daily temozolomide (TZM), and six cycles of adjuvant TZM – a median survival time of 14,6 months may be achieved in newly diagnosed GBM patients [2]. Resistance to TZM treatment, due to the activation of DNA repair proteins remains a major barrier to effective therapy [3] and high grade gliomas almost always recur. Salvage therapies at recurrence produce minimal improvement in 6-month progression-free survival [4]. Some alterations that govern GBMs has been outlined, the most frequent among them are LOH 10q, Phosphatase and Tensin homolog (PTEN) mutation/deletion and Epidermal Growth Factor Receptor (EGFR) amplification/overexpression [5]. EGFR has been found overexpressed in a number of GBMs [6] and has been used as a prime target for therapeutic intervention with inhibitory agents. However, several studies that have been conducted to evaluate the effectiveness of the EGFR inhibitors have shown that their use in unselected patients with malignant gliomas remains unproven [7-9]. Similarly, the use of inhibitors of other transduction pathways have been shown to be ineffective for the treatment of unselected patients suggesting that the inhibition of a specific pathway may result in the activation of a compensatory pathway that allows the tumour to survive. For these reasons novel therapeutic approaches are strongly needed.

Mitochondria-directed chemotherapy is emerging as a promising tool to combat apoptosis-resistant cancer cell proliferation [10-12]. Mitochondria are the cell energy producers and are essential for maintaining cell life; however, they also play a key role in cell death when their membranes become permeabilized. Mitochondrial membrane permeabilization includes either outer membrane permeabilization or inner membrane permeabilization (IMP). IMP produces the so called mitochondrial permeability transition (MPT) that compromises the normal integrity of the mitochondrial inner membrane which becomes freely permeable to protons leading to uncoupling oxidative phosphorylation [13]. The most accredited theory to explain the MPT is the opening of a multiprotein complex, the mitochondrial permeability transition pore (MPTP), located at the contact site between the inner and outer mitochondrial membranes. The composition of the MPTP is still unknown and results from the association of several proteins. Among them, the adenine nucleotide translocator (ANT), the voltage-dependent ion channel (VDAC), the translocator protein (TSPO), the hexokinase II (HKII) and ciclophyline D (CyP-D) are classically described [14].

Like many anti-cancer drugs, the effects of MPT-inducing agents are felt systemwide but fall most heavily upon cancer cells that present a switch to a predominant glycolitic metabolism which renders the mitochondrial transmembrane potential more instable. Moreover, a number of these agents induce MPT targeting MPTP components that are selectively up-regulated in cancer cells, such as the TSPO and ANT proteins [15-18], thus reinforcing the cancer selective action of the therapy. Agents reported to induce MPT targeting the MPTP, are able to induce cell death in several cells and some of them have also been reported to exert a mitochondria-mediated cytotoxic effect on glioma cells [19-23]. However, the activity of these compounds, as well as their mechanisms of action, have not been yet completely elucidated in high grade astrocytoma. The aim of this paper is to report a comprehensive analysis of the effects produced by a selected group of putative MPTP-targeting drugs (Betulinic Acid, Lonidamine, CD437, see [24] for a review) on a TZM-resistant GBM (IV WHO grade) cell line (ADF cells) that did not show EGFR amplification/overexpression and that has a hemizygous deletion of the PTEN gene.

Betulinic acid (BA), a natural product derived from the bark of the white birch tree [25], has been demonstrated to potently inhibit the growth of neuroectodermal tumors, such as neuroblastoma, medulloblastoma, and Ewing sarcoma cell lines [26] as well as several human carcinoma [27]. Although the protein target of BA is still unknown its effects on mitochondrial transmembrane potential are blocked by the MPTP inhibitor bongkrekic acid [22]. Lonidamine (LND) has been shown to induce apoptosis in drug-resistant cells [23] reducing aerobic glycolytic activity through the inhibition of mitochondrially-bound hexokinase (HK) which is present in large amounts in malignant cells [28,29]. This inhibition is probably operated through the interaction with the MPTP pore component ANT [30]. CD437 displays significant potential as a therapeutic agent in the treatment of a number of premalignant and malignant conditions [31]. The mechanism of action of CD437 is still poorly understood and it is probable that this drug acts on different cellular targets [32,33]. In vitro studies suggested that one of those targets is the ANT protein [30].

The data described in this paper will furnish information about the potential use of MPT-inducing agents for the treatment of high grade astrocytoma that resist conventional therapies or that do not show peculiar genetic alteration that can be targeted by specific drugs.

## Methods

### Drugs

BA (855057, Sigma Aldrich, St. Louis, MO), LND (L4900, Sigma Aldrich, St. Louis, MO), CD437 (C5865, Sigma Aldrich, St. Louis, MO), TZM (T2577, Sigma Aldrich, St. Louis, MO), Ciclosporin A (CsA, 30024, Sigma Aldrich, St. Louis, MO), CCCP (C2759, Sigma Aldrich, St. Louis, MO) have been purchased from SIGMA Aldrich. 20 mg/ml, 200 mM, 100 mM, 100 mM, 10 mM, stock solutions have been prepared in DMSO for BA, LND, TZM, CsA, and CD437 respectively. A 50 mM stock solution was prepared in ethanol for CCCP.

### Cell cultures, tumor and normal brain tissues

Human ADF GBM cell line (obtained from a WHO grade IV human GBM [34]), were maintained in standard culture conditions (37°C, 95% humidity, 5% CO_2_) in RPMI 1640 medium supplemented with 10% fetal bovine serum (FBS), 2 mM L-glutamine, 100 U/mL 7 penicillin and 100 μg/mL streptomycin. Two normal brain tissue samples and one WHO grade IV GBM sample were obtained from patients enrolled in a clinical-genetic protocol at Neurosurgery Unit of Livorno Hospital after the approval of the ethics review committee of Livorno City (SCS 2008-0019).

### Analysis of the expression of the EGFRvIII isoform

EGFR amplicons are often mutated and variant 3 (EGFRvIII) with deletion of exons 2 to 7 is the most frequent type. To analyze the presence of these variant, 1 μg of total RNA was retrotranscribed and amplified using the following primers:

Forward: 5'-GGGCTCTGGAGGAAAAGAAA-3'

Reverse: 5'-AGGCCCTTCGCACTTCTTAC-3'

that span from exon 1 to exon 8 [35] at the following amplification conditions: 2 minutes of initial denaturation at 95°C; 30 cycles including 95°C for 30 seconds, 55°C for 45 seconds and 72°C for 1 minute and 30 seconds; 5 minutes of final extention at 72°C.

RNA obtained from human normal brain tissues and from a WHO grade IV GBM known to express the EGFR variant 3 (Lena et al., manuscript in preparation) were also amplified as negative and positive controls respectively.

### Differential PCR

ADF cells, normal brain tissues and a grade IV glioma known to have EGFR amplicons and a hemizygous deletion of PTEN (Lena et al., manuscript in preparation) were screened for EGFR amplification and homozygous or hemizygous deletion of *PTEN *by differential PCR using genomic primers for *PTEN *exon 9 (forward 5'-AAACAGTAGAGGAGCCGTCA-3' and reverse 5'-GACTTTTGTAATTTGTGTATGCT-3') or EGFR exon 22 (forward 5'-CATCTGCCTCACCTCCACC-3' and reverse 5'-GCACACACCAGTTGAGCAG-3') together with primers for the internal allele dosage standard *GAPDH *gene from chromosome 12p (forward 5'-CCATCACTGCCACCCAGAA-3' and reverse 5'-TGCCAGTGAGCTTCCCGTT-3'). Differential PCR was performed using the Go-Taq PCR Kit (Promega, Madison, WI) starting from 50 ng of genomic DNA. To avoid unequal amplification efficiency of genomic PTEN or EGFR and of the internal standard GAPDH, different PCR conditions have been tested in brain tissue control samples to obtain amplification bands of equal intensity. According to this analysis, the amplification conditions were as follows:

For PTEN amplification: 95°C for two minutes, 30 cycles including 95°C for 30 seconds, 57°C for 45 seconds, 72°C for 30 seconds.

For EGFR amplification: 95°C for two minutes, 32 cycles including 95°C for 30 seconds, 55°C for 45 seconds, 72°C for 30 seconds.

For GAPDH amplification: 95°C for five minutes, 32 cycles including 95°C for 30 seconds, 53°C for 45 seconds, 72°C for 30 seconds.

The optimal number of cycles was established according to a stringent calibration process determining the log-linear phase of amplification for each gene.

After electrophoresis of the amplified products, each band was quantified using the ImageJ software [36] and the EGFR/GAPDH or PTEN/GAPDH ratio has been calculated. An EGFR/GAPDH ratio ≥ 2 was considered indicative of genomic amplification. A PTEN/GAPDH ratio ≤ 0.5 or ≤ 0.2 has been regarded as evidence of hemizygous or homozygous deletion respectively.

### PTEN mutation analysis

PTEN full length cDNA was amplified from ADF total RNA using the following primers:

Forward: 5'-ATGACAGCCATCATCAAAGAG-3'

Reverse: 5'-GACTTTTGTAATTTGTGTATGCT-3'

The amplification product was sequenced by automated fluorescent cycle sequencing (ABI).

### Karyological analysis

Chromosome preparations were made according to standard protocols. Human ADF cells were incubated with colchicine (0,05 μg/ml) for 3 h at 37°C. Cells, were harvested by trypsin, treated with hypotonic solution (0,05 M KCl) for 10 min at 37°C, and then fixed with Acetic Acid/Ethanol (1:3). After standard preparation, slides were stained with Giemsa (Carlo Erba). 100 metaphases were scored in three different slides to assess the chromosome number and aberration.

### Crystal violet assay

100000 ADF GBM cells were plated in 24 well plates. The following day the growth medium was replaced with fresh medium containing the drug at the final desired concentration and cells were left to grow for additional 24 hours. Cells were then washed twice with pre-warmed PBS and fixed in absolute cold methanol for 10 minutes at minus 20°. After two washes with room temperature PBS, cells remaining on the well plate were stained for ten minutes with a crystal violet solution (0.5% crystal violet, 20% methanol). After removal of the crystal violet solution, the plates were washed three times by immersion in a beaker filled with tap water. Plates were left to dry at 37° and 0.6 ml of crystal violet destaining solution (50% Ethanol, 0.1 M Sodium Citrate, pH 4.2) were then added to each well. Optical density was then measured reading the absorbance at 540 nm. Three wells for each drug concentration were measured; absorbance values were blank subtracted using as blank the optical density of wells containing only the growth medium. The percentage of the organic solvent, in which each drug was dissolved, never exceeded 1% (v/v) in the samples. We verified that this amount did not affect cell viability. The **I**nhibition **C**oncentration (IC50, the concentration of drug where 50% of cells die) for each compound was calculated by a sigmoidal dose-response curve, using the GraphPad Prism 4 program. To assess the specificity of the drug cyotoxic effect through the MPTP, ADF cells were first treated for 30 minutes with the MPTP-blocker CsA at 1 μM final concentration. After removal of the MPTP-blocker a new medium containing the MPT-inducing drugs at the desired concentration was added to the cells.

### Mitotic index

30000 ADF cells were plated in 24 well plates. The following day, cells were treated with drugs at the selected concentration and after additional 5 or 24 hours, adherent cells were detached, collected by centrifugation and resuspended in 40 μl of a glycerol, acetic acid, PBS (1:1:13) solution containing 5 μg/ml of the *bis*-benzimide Hoechst 33342 (Invitrogen, H21492, Carlsbad, CA). Cells were treated with 0.05 μg/ml colchicine for 3 hours before collection. Two 5 μl aliquots of cell suspension for each sample were spotted on a glass slide and allowed to dry. The number of mitotic figures was counted under the fluorescence microscope. Two 10 μl aliquots for each sample were used to count the number of total cells with a hemocytometer. For each treatment, the mitotic index (mitotic figures/total cells) was calculated in 3 replicate wells. Two independent experiments were performed. To assess the specificity of the drug cytostatic effect through the MPTP, ADF cells were first treated for 30 minutes with the MPTP-blocker CsA at 1 μM final concentration. After removal of the MPTP-blocker a new medium containing the MPT-inducing drugs at the desired concentration was added to the cells.

### Evaluation of mitochondrial potential by (JC1) staining assay

5,5',6,6'-tetrachloro-1,1',3,3'-tetraethylbenzimidazolylcarbocyanine iodide (JC1; Invitrogen, T3168, Carlsbad, CA) is a cationic dye that exhibits potential-dependent accumulation in mitochondria, indicated by a fluorescence emission shift from green (~525 nm) to red (~590 nm). Consequently, mitochondrial depolarization is indicated by a decrease in the red/green fluorescence intensity ratio and can be quantified by using both flow cytometry or fluorescence microscopy [37]. To evaluate the mitochondrial depolarization induced by drug treatment, we plated 10000 ADF cells in 96 well plates. The following day cells were stained for 10 minutes in medium containing JC-1 at the final concentration of 50 μg/ml. After removal of JC-1, a new medium containing the drug, at the desired final concentration, was added to the cells. 3, 6 and 24 hours after the treatment pictures were taken under an Axiovert fluorescent microscope (Zeiss) using the filter set 10, 488010-0000 (Zeiss) (excitation 450–490: emission 515–565). Pictures were then split in the RGB channels (red and green) and analyzed by using the program ImageJ [36]. The ΔΨ **I**nihibition **C**oncentration (ΔΨ IC50, the concentration of drug where 50% of ΔΨ is dissipated) was calculated using the GraphPad Prism 4 program. To assess the specificity of drug-induced depolarization through the MPTP, JC1-loaded ADF cells were first treated for 30 minutes with the MPTP-blocker CsA at 1 μM final concentration. After removal of the MPTP-blocker a new medium containing the MPT-inducing drugs, at the desired concentration was added to the cells.

### Assessment of cell death modality

#### - Hoechst uptake, propidium iodide incorporation and acetomethoxy derivate of calcein staining assays

20000 cells were plated in 96 well plates. The following day, cells were treated with drugs at the selected concentration and after additional 24 or 48 hours were stained with 5 μg/ml Hoechst 33342, 2 μg/ml Propidium iodide (PI, Sigma-Aldrich, 81845, St. Louis, MO) and 1 μM acetomethoxy derivate of calcein (calcein AM, Sigma-Aldrich, C1359, St. Louis, MO) for 10 minutes at 37°C. After staining, both floating and adherent cells were collected and analyzed using a hemocytometer under a fluorescence microscope. Cells that, independently from calcein staining, avidly incorporated the Hoechst dye and showed typical morphological features such as chromatin condensation and margination, were considered apoptotic cells according to [38]. Frequently, late apoptotic cells were also PI positive due to a secondary necrotic process that generally takes place in cultured apoptotic cells. The ratio between apoptotic cells and total cells (A/T ratio) was calculated. Cells that were calcein negative and PI positive and that did not show the typical nuclear apoptotic alterations were considered necrotic cells. The ratio between necrotic and total cells (N/T ratio) was calculated. Cells that were hoechst 33342 and PI negative and that showed a strong calcein staining were considered live cells. The ratio between live and total cells (L/T ratio) was calculated. The A/T, N/T and L/T ratios were evaluated in three wells for each experimental condition; cells detached from each well were counted in duplicate. To assess the specificity of the drug apoptotic effect through the MPTP, ADF cells were first treated for 30 minutes with the MPTP-blocker CsA at 1 μM final concentration. After removal of the MPTP-blocker new medium containing the MPT-inducing drugs at the desired concentration, was added to the cells.

#### - Annexin V binding assay

Based on the phenomenon that phospholipids (PS) are exposed during apoptosis and on the ability of annexin V to bind to PS with high affinity, we used annexin V to detect apoptosis. 15000 cells were plated in 96 well plates. The following day, cells were treated with the drugs at the desired concentration and after an additional 24 hours, we analyzed the annexin V-positive/PI-negative cells using the Annexin V-FITC Fluorescence Microscopy Kit (BD Biosciences, Franklin Lakes, NJ) following manufacturer's instructions.

#### - Detection of acidic vesicular organelles (AVOs)

As a marker of autophagy, the appearance and volume AVOs was visualized by acridine orange staining [39]. Briefly, 20000 ADF cells were seeded in 96 well plates. The following day, cells were treated with drugs at the selected concentration and after 6, 24 or 48 hours were incubated in serum-free medium containing 1 μg/ml acridine orange for 15 minutes at 37°C. The acridine orange was removed and fluorescent micrographs were taken using an inverted fluorescent microscope. The cytoplasm and nucleus of the stained cells fluoresced bright green, whereas the acidic autophagic vacuoles fluoresced bright orange. In order to carry out a specificity control cells were treated with 200 nM bafilomycin A1 for 30 minutes before the addition of acridine orange to inhibit the acidification of autophagic vacuoles.

## Results

### Genetic characterization of the human glioma ADF cells

In order to characterize the cellular model system to test the selected MPT-inducing agents, some karyological and genetic aspects of ADF cells (especially the the most frequently described genetic aberration in gliomas) were analyzed. Karyological studies revealed that ADF cells are aneuploid with a mean number of chromosomes for metaphase of 58 ± 5. Moreover several chromosomal abnormalities such as double minutes and single or double chromatid gaps or breaks were detected. Interestingly, about 50% of ADF cells showed a single minute frequently associated with a medium-size sub-metacentric chromosome (data not shown).

**Figure 1:**
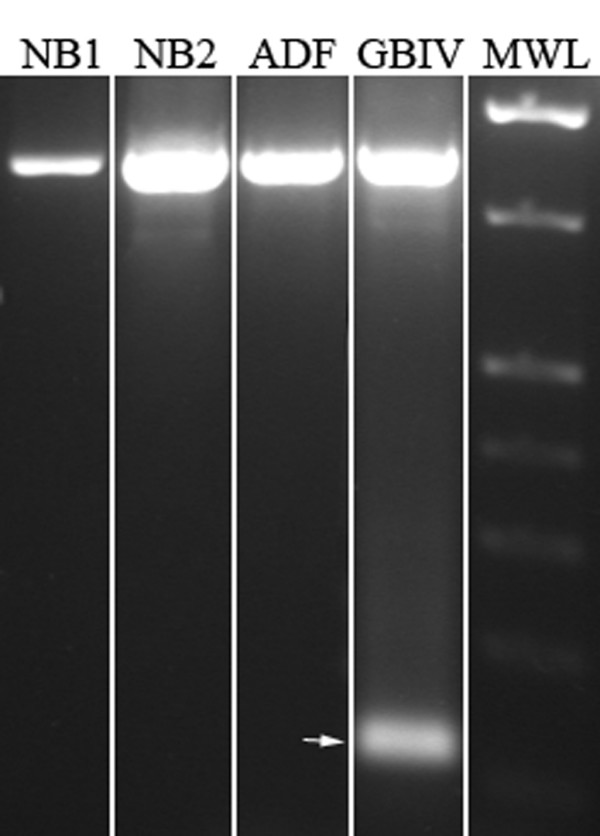
**Analysis of EGFR variant III expression**. EGFRvIII was not expressed in ADF cells. Arrow indicates EGFRvIII (128 bp) amplification band. NB1, normal brain tissue sample 1; NB2, normal brain tissue sample 2; GBIV, WHO grade IV glioblastoma; MWL, molecular weight ladder.

**Figure 2:**
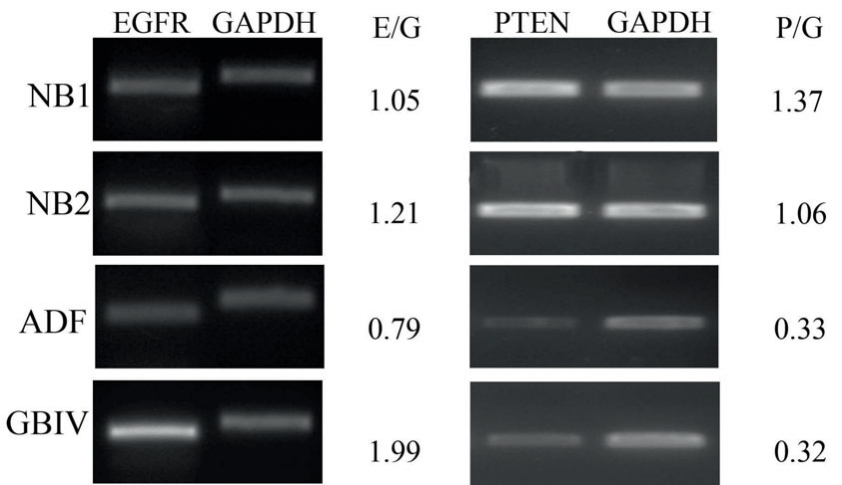
**Differential PCR**. ADF cells did not show EGFR amplification and have a hemizygous deletion of the chromosome 10q region containing the PTEN locus. Amplification bands of representative experiments are depicted for each gene in the analized samples. NB1, normal brain tissue sample 1; NB2, normal brain tissue sample 2; GBIV, WHO grade IV glioblastoma; E/G, mean ratio of densitometry values of EGFR and GAPDH amplification bands. P/G, mean ratio of densitometry values of PTEN and GAPDH amplification bands.

As demonstrated by EGFR transcript amplification, by RT-PCR assay, ADF cells and normal brain tissue did not express the EGFRvIII variant that is visible in a grade IV glioblastoma sample used as positive control (Fig 1). Accordingly, ADF cells did not show EGFR genomic amplification as demonstrated by densitometry analysis of the differential PCR assay. On the contrary an EGFR/GAPDH ratio ≥ 2 was obtained in a grade IV glioblastoma sample, known to have EGFR amplicons, that we used as positive control (Fig. 2).

Sequence analysis of PTEN cDNA, isolated from ADF cells, did not reveal mutations. However, differential PCR analysis performed using PTEN exon 9 directed primers, revealed a PTEN/GAPDH ratio of 0.3 indicating an hemizygous deletion of PTEN. As expected a PTEN/GAPDH ratio close to 1 was found in normal human brain tissues and a 0.3 ratio was found in a grade IV GBM sample, known to have PTEN hemizygous deletion that we used as a positive control (Fig. 2).

### MPT-inducing drugs affect TZM-resistant glioma cell viability and dissipate the mitochondrial transmembrane potential through the modulation of MPTP opening

**Figure 3:**
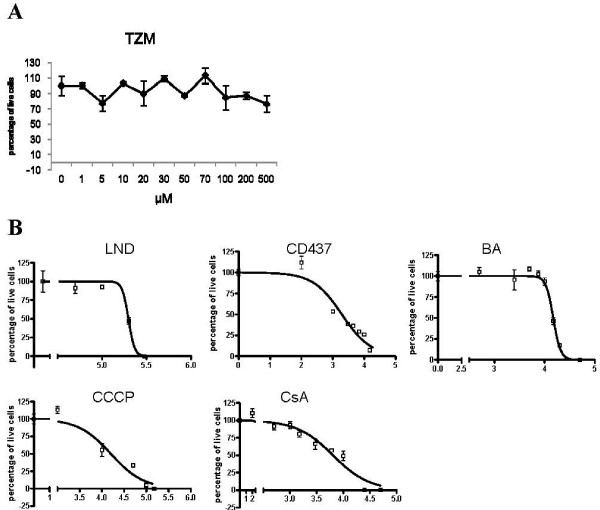
**Effect of CD437, LND, BA, TZM, CCCP and CsA treatment on ADF cell viability as assessed by crystal violet assay**. ADF cells are resistant to TZM and their viability is affected by CD437, LND and BA. (A) The Graph indicates the dose dependent effect on cell viability measured 24 hours after the treatment with TZM. (B) Sigmoidal dose-response curve of the effect on cell viability measured 24 hours after the treatment with LND, CD437, BA, CCCP and CsA. Each value has been normalized versus the vehicle treated control to which an arbitrary value of 100% has been assigned. Each point is the mean of two independent experiments performed in triplicate.

Crystal violet staining assay was performed to test the reduction in cell viability produced by TZM and by the selected MPTP-targeting drugs on ADF glioma cells. As shown in Figure 3, ADF cells were unaffected by TZM, 24 hours after treatment. On the contrary, LND, CD437 and BA affected ADF viability in a dose dependent manner, 24 hours after treatment. IC50 values were 13 ± 2; 6 ± 3 and 240 ± 10 for BA, CD437 and LND respectively. CCCP was used as a positive control. The treatment with the well known MPTP blocker CsA did not produce a reduction in cell viability at the concentration of 1 μM at which it will be used in the following experiments (see below).

**Figure 4:**
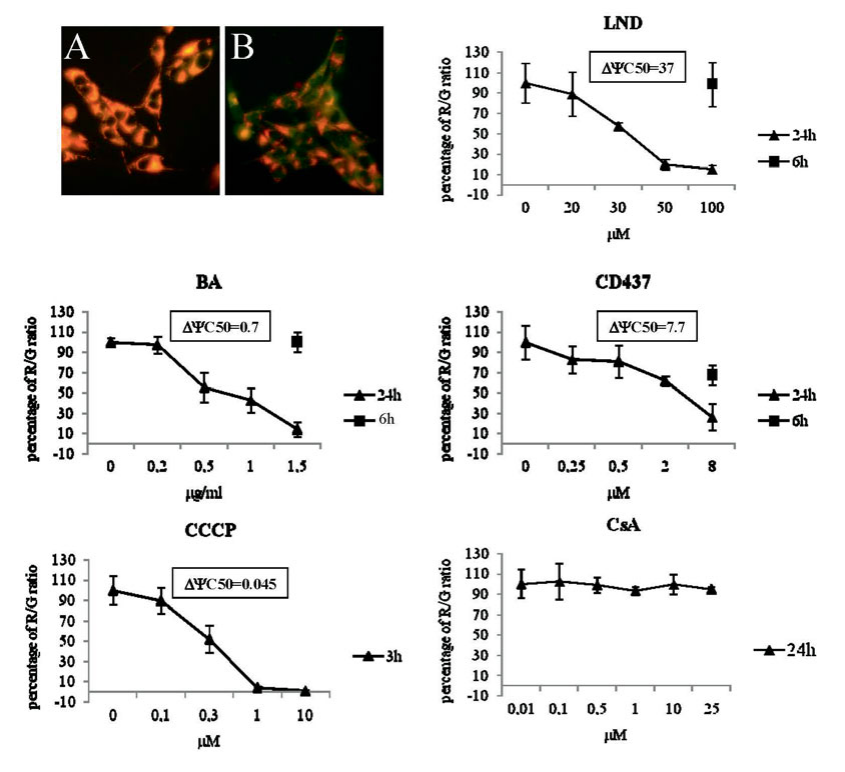
**Effect of CD437, LND, BA, CCCP and CsA treatment on mitochondrial transmembrane potential as assessed by JC1 staining**. CD437, LND and BA treatment produces a dose dependent ΔΨ dissipation. (A) Vehicle treated cells stained with JC1. (B) ADF cells treated with BA at the ΔΨ IC50 dose and stained with JC1. Graphs indicate the dose dependent ΔΨ dissipation expressed as Red/green (R/G) fluorescence ratio. Each value has been normalized versus the R/G ratio of the vehicle treated control to which an arbitrary value of 100% has been assigned. Each point is the mean of two independent experiments performed in triplicate.

**Figure 5:**
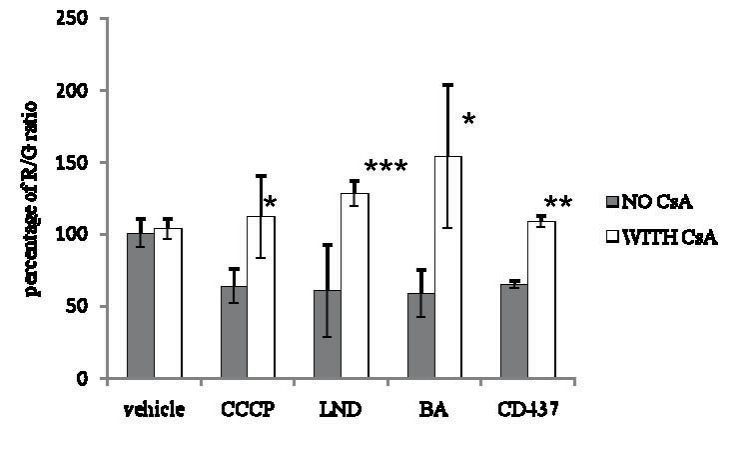
**Effect of CsA pre-treatment on ΔΨ dissipation produced by CCCP, CD437, LND and BA treatment**. CsA treatment prevents CD437-, BA- and LND-induced ΔΨ dissipation. Each value has been normalized versus the R/G ratio of the vehicle treated control without CsA to which an arbitrary value of 100% as been assigned. R/G ratios have been calculated 24 hours after treatment at the IC50 dose. Each bar is the mean of two experiments performed in triplicate. The R/G ratios of drug treated samples normalized versus the R/G ratio of the vehicle treated control were compared with the R/G ratio of CsA+drug treated samples normalized versus the R/G ratio of the CsA+vehicle treated control using the unpaired *t*-test. *P < 0.05; **P < 0.01; ***P < 0.001.

To test whether the reduction of viability produced by LND, CD437, and BA was the result of MPT induction, the ΔΨ dissipation as a consequence of a 6 or 24 hour-long treatment with the drugs was analyzed. As shown in Figure 4, all the selected drugs were able to produce a sustained ΔΨ dissipation 24 hours after treatment. ΔΨ IC50 are also reported in Figure 4. As expected a 24 hour-long treatment with the MPTP-blocker CsA did not produce ΔΨ dissipation. ΔΨ dissipation was also evaluated 6 hours after the treatment using the highest concentration tested in the 24 hour-long exposure. Only CD437 was able to produce an early significant ΔΨ dissipation. A 3 h treatment with the uncoupling agent CCCP was used as positive control. To determine whether the drug-induced MPT was mediated by the opening of MPTP a pre-treatment with the MPTP-blocker CsA was performed prior to the treatment with the selected drugs. As shown in Figure 5, CsA treatment alone did not show any effect on ΔΨ, while CsA pretreatment totally abolished the ΔΨ dissipation effect triggered by CCCP, LND, CD437 and BA.

**Figure 6:**
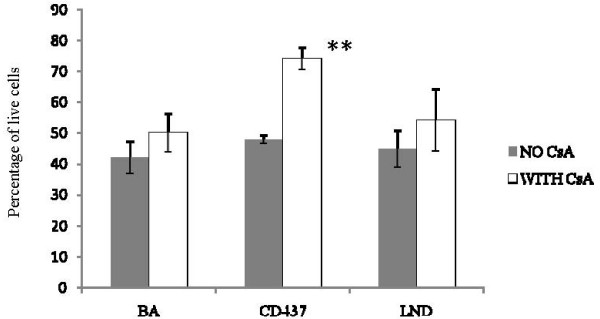
**Effect of CsA pre-treatment on viability reduction produced by CD437, LND and BA treatment**. CsA treatment protects ADF cells from CD437 effects and has no influence on the reduction in cell viability produced by BA and LND. Each value has been normalized versus the vehicle treated control to which an arbitrary value of 100% as been assigned. Cell viability has been measured by crystal violet assay 24 hours after the treatment at the IC50 dose. Each bar is the mean of two experiments performed in triplicate. The absorbance values of the drug treated samples were compared with those of the vehicle treated control using the unpaired *t*-test. **P < 0.01.

With the aim of understanding whether MPT induction through the MPTP plays a leading role in the reduction in cell viability produced by the selected drugs, the capability of CsA to protect ADF cells from the effects of MPT-inducing drugs on cell viability was analyzed. As shown in Figure 6, the effect of CD437 on cell viability, evaluated by crystal violet staining, was significantly reduced when a pre-treatment of 30 minutes was performed with the MPTP-blocker CsA at 1 μM, a concentration that does not alter cell viability after 24 hours of continuous treatment (Fig. 3). On the contrary, the effects produced by LND and BA treatment at the IC50 dose were not significantly affected by the CsA pre-treatment.

### MPT-inducing drugs act as both cytostatic and cytotoxic compounds on ADF cells

**Figure 7:**
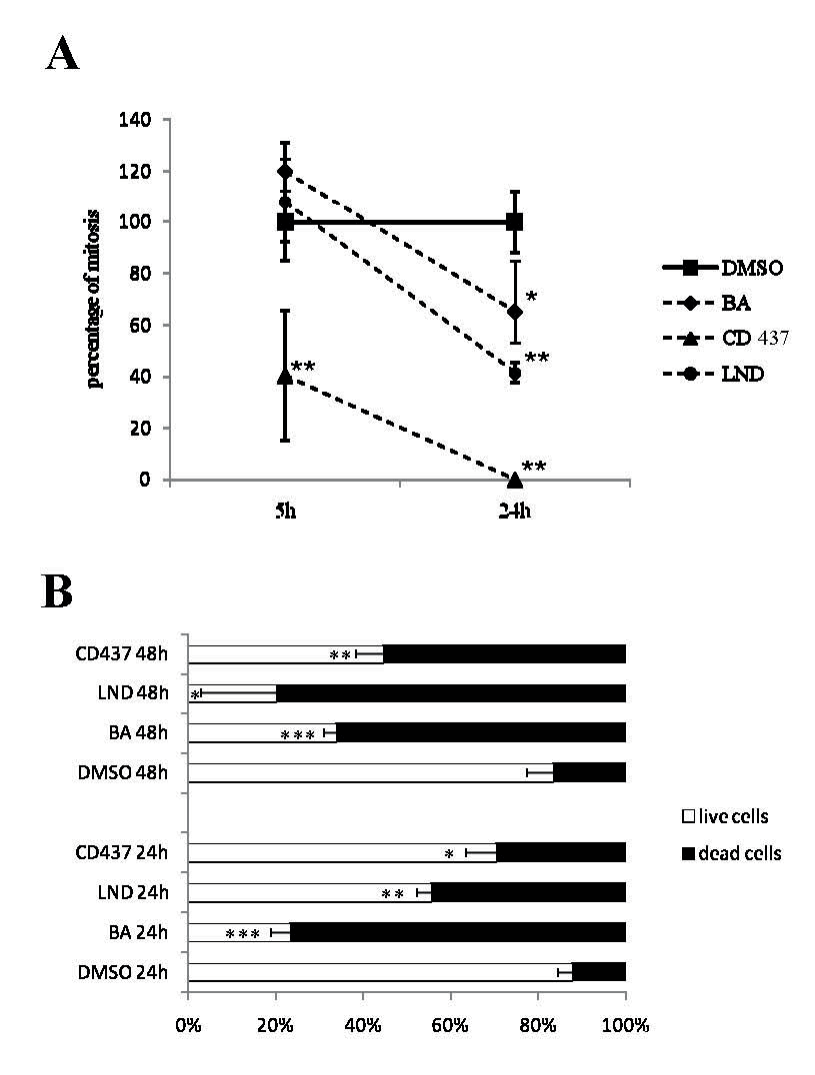
**Cytostatic and cytotoxic effects of CD437, LND and BA treatment on ADF cells**. CD437, BA and LND exert both cytostatic and cytotoxic effects on ADF cells. (A) Mitotic index analysis. Each value has been normalized versus the respective vehicle treated control to which an arbitrary value of 100% as been assigned. Each point is the mean of two independent experiments performed in triplicate. The mitotic indexes of drug treated samples were compared with those of the vehicle treated controls using the unpaired *t*-test *P < 0.05; **P < 0.01. (B) Calcein AM staining assay. Each bar indicates the percentage of live and death cells and is the mean of two experiments performed in triplicate. The number of live cells counted in the drug treated samples was compared to that of the vehicle treated controls using the unpaired *t*-test. *P < 0.05; **P < 0.01; ***P < 0.001.

Mitotic indexes have been calculated to evaluate the cytostatic effect produced on ADF cell proliferation by treatment with the selected drugs at the IC50 dose. As shown in Figure 7A, BA and LND produced a significant reduction of the mitosis number, 24 hours after the treatment. Interestingly, CD437 produced an early antiproliferative effect 5 hours after the treatment that resulted in the complete absence of mitosis, 24 hours after treatment. Reduction of mitoses produced by CD437 proved insensitive to CsA pre-treatment.

Calcein AM staining was used to evaluate the cytotoxic effect produced by the selected drugs at the IC50 dose. Calcein AM is transported through the cellular membrane into live cells, where intracellular esterases remove the acetomethoxy group allowing the molecule to bind calcium and to produce a strong green fluorescence. As dead cells lack active esterases, only live cells are labeled. As shown in figure 7B the number of live calcein positive cells was significantly reduced 24 and 48 hours after treatment with the selected drugs at the IC50 dose.

### MPT-inducing drug treatment leads to apoptosis

**Figure 8:**
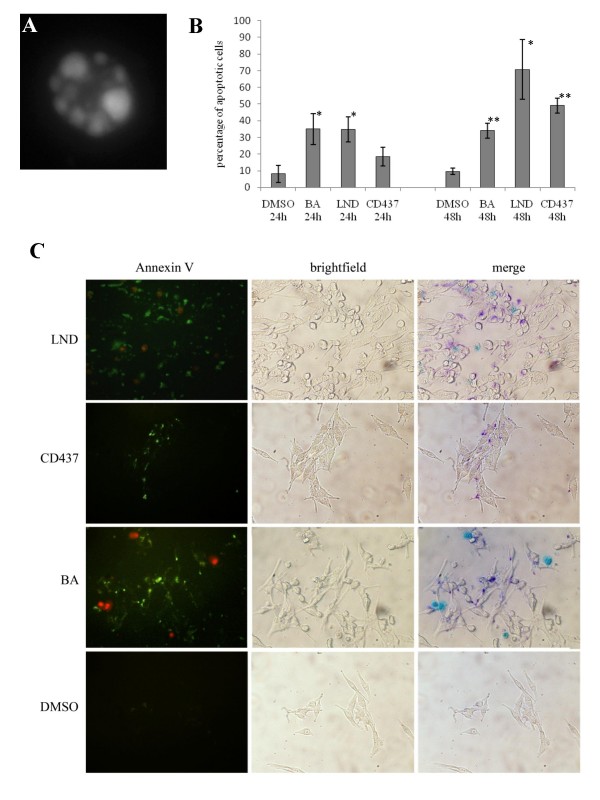
**Assessment of apoptotic cell death**. CD437, BA and LND induce apoptosis of ADF cells. (A) An apoptotic nucleus. (B) Percentage of apoptotic cells (assuming as 100% the number of total cells) in drug treated samples and in vehicle treated controls 24 and 48 hours after treatment. The number of apoptotic cells counted in the drug treated samples was compared with that of the vehicle treated controls using the unpaired *t*-test. *P < 0.05; **P < 0.001. (C) A representative example of annexin V binding assay. Several cells show annexine V cross reactivity on their membranes in CD437, BA and LND treated samples. In the merged panel Annexin V and PI signals appear purple and cyan respectively.

Owing to changes in membrane permeability, early apoptotic cells show an increased uptake of Hoechst 33342 compared with live cells [38]. This feature was used to assay the ability of LND, BA, and CD437 to produce apoptotic cell death. In our analysis we considered as apoptotic cells those cells that showed strong Hoechst 33342 staining and typical apoptotic nuclear morphology (Fig. 8A). As indicated in Figure 8B in CD437, BA, and LND treated cells a higher number of Hoechst positive nuclei was observable with respect to vehicle treated controls 24 and 48 hours after the treatment at the IIC50 dose. Interestingly, the CsA pretreatment reduced to a half the number of apoptotic cells counted after CD347 treatment. In this assay, we discriminated between dead (necrotic) and apoptotic cells by adding the membrane impermeable DNA dye PI simultaneously to the cells. Those cells that were PI positive, calcein AM negative and that showed a normal nuclear morphology we considered to be necrotic. A few necrotic cells were counted in BA and LND treated cells 24 and 48 hours after the treatment (data not shown). To confirm the ability of the selected drugs to induce apoptotic cell death, we also evaluated the PS externalization through Annexin V binding 24 hours after treatment with the selected drugs at the IC50 dose. All the analyzed drugs showed a significant increase in Annexin V reactivity with respect to vehicle treated controls (Fig. 8C). In addition, the majority of Annexin V positive cells did not show PI staining.

**Figure 9:**
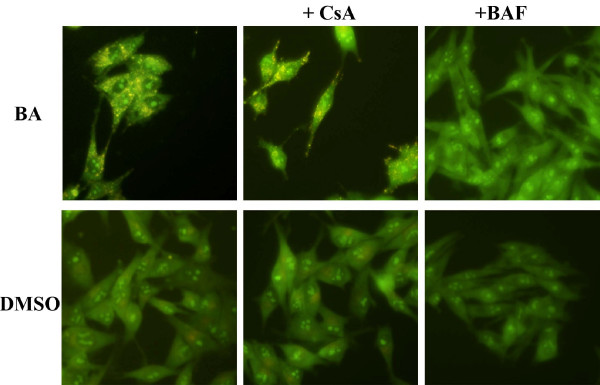
**Assessment of AVOs formation by acridine orange staining**. BA induces autophagy in ADF cells. A representative example of acridine orange stained cells. Bright orange granules are evident in BA and CsA+BA treated cells. As specificity control, cells treated with 200 nM bafilomycin A1 (BAF) for 30 minutes before the addition of acridine orange do not show AVOs formation.

The ability of the selected drugs to induce autophagic cell death was also analyzed. Autophagy is characterized by the development of acidic vesicular organelles (AVO), which is measured by vital staining of acridine orange. AVO positive cells were not detectable 6, 24 and 48 hours after the treatment with CD437 and LND either at the IC50 or higher doses. However, several AVO-positive cells were detectable 6, 24 and 48 hours after treatment with BA (Fig. 9). The number and brightness of BA induced AVO were not reduced in the presence of CsA (Fig. 9).

## Discussion

The aim of this paper is to propose MPT inducing drugs as adjuvants in chemotherapy protocols for the treatment of high grade astrocytoma.

Several ground-breaking therapeutic approaches, based on inhibitors of tumor-specific hyperactivated transduction pathways, are emerging for glioma treatment. However, the outcome of this kind of treatment is strictly dependent upon the gene expression/protein activation profile of each malignant glioma. Thus, these inhibitors are not recommended for unselected malignant glioma patients leaving a large part of them without an alternative to TZM.

Gliomas are a very diverse and complex group of neoplasms with a pronounced genetic heterogeneity. Thus the in vitro analysis of the effects produced by a putative chemotherapeutic agent should be analyzed taking into account the type of genetic category in which the model cell line fit into and, consequently, translate in vivo the obtained data in accordance with the genetic mutations that govern GBM. To this aim we characterized ADF cells in accordance with the most frequent genetic aberrations that are documented in glioblastoma. ADF cells show a high kariologycal variability with several chromosomal aberrations including recurrent minute chromosomes that are frequently detected in tumor cells and are indicative of gene amplification. ADF cells did not show EGFR amplification as demonstrated by both the absence of the EGFR variant III and the low EGFR/GAPDH ratio obtained in the differential PCR assay. This feature excludes ADF cells as targets for the inhibitors of EGFR. Although ADF express a wild-type form of PTEN, differential PCR assays indicated a hemizygous deletion of the chromosome 10q region containing the PTEN locus suggesting a reduced PTEN expression leading to a strongly deregulated cell growth. Interestingly, we demonstrated that ADF cells are unaffected by high TZM concentrations, suggesting that they have developed TZM resistance as often happens in vivo at early recurrence of glioblastoma tumors. This data are consistent with a previous report that demonstrated that ADF cells are resistant to carmustine treatment [40]. For these reasons, ADF cells represent the elective model system of high grade astrocytoma cells, untreatable with standard and trial therapies, where to test the effects of mitochondria-damaging agents.

Here, we firstly tested the ability of BA, LND and CD437 to affect ADF cell viability in a dose dependent manner 24 hours after treatment by using the crystal violet assay. All the tested compounds were able to reduce cell viability with respect to vehicle treated controls. The ability of BA to reduce cell viability is particularly interesting since, for glial tumors, previously published results are conflicting. In fact, although this drug was originally claimed to induce apoptosis in several cancer cell lines [27], very high concentrations of BA (as high as 50 μg/ml) were needed to detect signs of apoptosis in quite a few malignant glioma cell lines [41]. In this paper we demonstrate that ADF cells are highly affected by BA treatment and that a 24 hour treatment with 12.5 μg/ml is able to produce a 50% reduction in cell viability. This finding suggests that still unknown genetic features make different glioma cell lines differently vulnerable to BA treatment.

We also demonstrated that LND, BA and CD437 are able to induce a potent ΔΨ dissipation mediated by MPTP as demonstrated by the ability of CsA to totally abolish LND-, BA- and CD437-mediated MPT induction. Among the selected compounds, CD437 proved more efficient in the ΔΨ dissipation being able to produce a very early mitochondrial damage. Moreover, the effects triggered by this compound on cell viability were significantly reduced as a consequence of a CsA pre-treatment, indicating that ΔΨ dissipation is a leading event for cell viability reduction produced by CD437. CD437 mechanism of action is still poorly understood and this finding suggests that the primary and early mitochondrial damage could be responsible for the other effects described for this drug [33,41-44]. On the contrary, ΔΨ dissipation is probably only a concurrent event in cell viability reduction produced by LND and BA. This hypothesis is also confirmed by the discrepancy between the very low LND and BA dose necessary to produce ΔΨ dissipation and the highest dose of these drugs useful to induce viability reduction. Moreover, this finding is consistent with the role proposed for LND that being thought to inhibit glycolysis by inactivation of the mitochondrially bound hexokinase, it could affect cell viability inhibiting the exclusive glycolitic cancer cells metabolism [45].

Crystal violet assay allows us to evaluate the number of cells that remain adherent to the well plate after the treatment. However, this assay does not give any information about the mechanism responsible for the reduction in viability. Several hypotheses could be therefore assumed, one of which is that the compounds could act as cytotoxic and/or cytostatic agents. To test a hypothetical cytostatic activity of the selected compounds we evaluated the mitotic index of cells treated at the IC50 dose 5 and 24 hours after treatment. For LND and BA a reduction in the mitotic index could be observed only 24 hours after the treatment, while CD437 proved very active in inhibiting cell proliferation, and an early reduction in the number of mitoses can be observed 5 hours after the treatment and no mitosis could be counted 20 hours later. The antiproliferative effect of CD437 was insensitive to CsA pre-treatment indicating that CD437 induced cell-cycle arrest is not mediated by MPTP.

We also evaluated the cytotoxic activity of the selected drugs. Firstly we evaluated the number of live and death cells using as samples both adherent and floating ADF cells 24 and 48 hours after the treatment at IC50 dose. For all the compounds a significantly higher number of death cells could be detected in comparison with the vehicle treated controls 24 and 48 hours after the treatment. In particular, a very high number of dead cells were observed in BA treated samples starting from 24 hours after the treatment while a noticeable high number of dead cells could be observed in CD437 treated samples only 48 hours after treatment. Taken together the data obtained from both the calcein AM staining and mitotic index, allow us to suggest that LND and BA have a primary cytotoxic effect and that the reduction in the proliferation rate observed 24 hours after the treatment could be a consequence of cell stress. On the contrary, CD437 causes an early proliferation arrest that may account for a consistent part of the reduction in cell viability monitored by crystal violet assay. Only in a second phase, do cell cycle arrested cells undergo a death process.

All the tested compounds produce their citotoxic effect inducing apoptotic cell death as demonstrated by Hoechst uptake and annexin V binding assays. Only a few necrotic cells were detected in LND and BA treated cells excluding necrosis as a relevant death pathway induced by these compounds. The number of apoptotic cells in CD437 treated samples was only slightly higher than that counted in vehicle treated samples 24 hours after the treatment, confirming that the primary effect produced by CD437 is the cell cycle arrest. Later on, a higher and significant number of apoptotic cells was observed in CD437 treated samples with respect to controls. CsA pre-treatment strongly reduced the number of apoptosis in CD437 treated samples indicating that the cell cycle-arrested cells produced by the treatment with this drug underwent apoptotic cell death primarily through MPT induction.

We also assayed the ability of the selected compounds to produce autophagic cell death by using the acridine orange staining. CD437 and LND were not able to induce AVOs formation in the cell cytoplasm. On the contrary, BA was able to induce AVOs formation very early after treatment. BA capability to induce AVOs was not inhibited by CsA pre-treatment suggesting that BA can also produce a cytotoxic effect through a MPTP-independent mechanism thus explaining the CsA insensitive cell viability reduction produced by this drug. The coexistence of different death mechanisms in BA treated cells could also account for the discrepancy between the extraordinarily high number of dead cells observed with calcein AM staining and the relatively lower number of apoptotic cells counted by Hoechst incorporation assay.

It can be expected that the treatment with compounds that lead to ΔΨ dissipation produces a stereotyped cell response. Actually, data obtained in this paper confirm several previous reports that describe different cell responses to the administration of MPT-inducing drugs. To explain this diversity it has to be taken into account that the different compounds produce MPT through different pathways by acting on still not well defined mitochondrial targets. As a consequence, the timing and intensity of MPT are extremely variable. This feature, together with the possible action of the MPT-inducing agents on other cellular targets could explain the variable cell response.

## Conclusion

In conclusion, mitochondrial damaging agents have the potential to be included in therapeutic protocols as adjuvant to radiotherapy or TZM treatment. The potential value of LND has been previously evaluated for the treatment of malignant gliomas [46,47]. In particular, a phase II clinical trial suggested a limited, but clear therapeutic activity of LND in association with radiotherapy as a first line treatment and in association with Lomustine (CCNU) at recurrence. The ability to reduce viability of TZM-resistant cells encourages re-evaluation of the use of LND in chemotherapy protocols in new combinations with other cytotoxic and/or antiproliferative drugs. We were not able to find any published data about clinical trials including CD437 and BA for glioblastoma treatment. Our data as well as other literature [22,26,41,48-51] confirm the antitumor activity of the novel retinoid and of the white birch tree extract on high grade astrocytoma cells, encouraging their use in clinical protocols.

## Competing interests

The authors declare that they have no competing interests.

## Authors' contributions

AL: carried out the JC1 staining assays, the apoptosis and autophagy assays, participated in the experimental design of the study. MR: carried out the mitotic index and the calceine AM staining assays, participated in the experimental design of the study. AS: participated in the design of the study, helped to draft the manuscript. BB: carried out the crystal violet assays. DV: carried out the molecular genetic studies. VS: carried out the karyological studies. RA: collected human normal brain and human glioma samples, helped to draft the manuscript. LB: collected human normal brain and human glioma samples. RG: collected human normal brain and human glioma samples. VG: participated in the design of the study, helped to draft the manuscript. LR conceived the study, coordinated the experimental design of the study, collected and analyzed the data, drafted the manuscript. All authors read and approved the final manuscript.
